# A study on the blended learning effects on students majoring in preschool education in the post-pandemic era: An example of a research-method course in a Chinese university

**DOI:** 10.3389/fpsyg.2022.962707

**Published:** 2023-01-11

**Authors:** Weiguaju Nong, Jian-Hong Ye, Pengfei Chen, Yi-Sang Lee

**Affiliations:** ^1^Dhurakij Pundit University, Bangkok, Thailand; ^2^School of Education, Guangxi University of Foreign Languages, Guangxi, China; ^3^Faculty of Education, Beijing Normal University, Beijing, China; ^4^Department of Industrial Education, National Taiwan Normal University, Taipei, Taiwan

**Keywords:** belief-action-outcome, blended learning, flipped classroom, learning engagement, O-PIRTATD/S, post-pandemic era, self-confidence, self-efficacy

## Abstract

The world has gradually entered the post-pandemic era. Although the pandemic has been slowing down, it still has a strong impact on the education scene. Thus, how to provide students with an effective and flexible learning style is currently an important educational issue. This study focused on the implementation of effective teaching to improve the learning effects based on these special circumstances. To ensure a realistic teaching situation, an experiment of blended learning was conducted in a university in the Guangxi Zhuang Autonomous Region of China for a short-term study, based on the consideration of pandemic supervision and control. In this experiment, a single-group quasi-experimental design method, using the extended O-PIRTATD/S model, was adopted in a research-method course designed for students majoring in preschool education. This research-method course was an 8-week flipped course, where the first 4 weeks were online teaching and the last 4 weeks were taught offline, thus combining as a blended learning course. A total of 115 active participants were recruited for this study. Based on the theoretical framework of the belief-action-outcome (BAO) model, five research hypotheses were proposed with the aim of constructing a learning behavior pattern based on cognitive beliefs which could be verified by structural equation modeling. The results of the study were concluded as follows: 1. Academic self-efficacy had a positive impact on learning engagement, but had a negative impact on cognitive load; 2. Cognitive load did not have a significant impact on learning engagement; 3. Learning engagement had a positive impact on the enhancement of academic self-confidence; and 4. Cognitive load had a negative impact on the enhancement of academic self-confidence.

## Introduction

COVID-19 broke out at the end of 2019 and spread rapidly around the world. It had a strong negative impact on the global economy, education, and society (Hao et al., 2022). Although we have now entered the post-pandemic age when people are trying to coexist with COVID-19, the impact on the education system is still severe and critical (Setiawan et al., 2021). Since the pandemic is continuing and uncertainty remains in the post-pandemic era, it is necessary to adopt appropriate measures to deal with the rapidly spreading crisis (Singh et al., 2021). Although pandemic prevention measures in many regions have been gradually loosened in the post-pandemic era, it is nevertheless difficult to have a daily routine as before. During this transitional period, COVID-19 is still having a profound impact on some countries and regions, and it is difficult to eradicate the virus in a short period of time. When people coexist with COVID-19, pandemic prevention and control inevitably become the routine. Therefore, while the order of normal life is gradually recovering, governments are still responding appropriately whenever necessary according to the unpredictable impact of COVID-19 and the severity of the pandemic measures, including in the education system.

Despite the gradual resumption of offline teaching, a full resumption of traditional face-to-face teaching is unlikely due to the unpredictability of COVID-19 (Al-Fodeh et al., 2021). In February 2022, there was an outbreak of COVID-19 in the Guangxi Zhuang Autonomous Region of China, and some colleges and universities in the region immediately switched from face-to-face to online teaching in order to cope with the impact of the pandemic. Due to various uncertainties in the post-pandemic era, teaching methods were rapidly adjusted considering the prevention and control of the pandemic. Therefore, as Al-Fodeh et al. (2021) stated, blended learning is the preferred teaching method in the post-pandemic era, since it can flexibly switch between online and offline teaching depending on the impact of the pandemic.

Nevertheless, Hong et al. (2021) pointed out that during the pandemic prevention and control blockade, most online courses in China were conducted in the form of lectures by teachers, while students watched and learned. No matter how the teaching format was adjusted, students should not be regarded as passive recipients of knowledge (Hunter-Doniger and Sydow, 2016). The traditional teaching-based teaching method was not coping with the learner-centered education policy of this generation. Therefore, even if they were affected by the pandemic and switched to online teaching or mixed teaching, teachers should still actively adopt a student-centered teaching method.

Among many teaching methods, the flipped classroom can make students responsible for their own learning rhythm and learning process (Huang et al., 2022). In recent years, the flipped classroom model has had a profound impact on higher education teaching and learning (Al-Samarraie et al., 2020). Therefore, the flipped classroom model was adopted for the curriculum design in this study. Enfield (2016) pointed out that in order to promote active learning, more attention should be paid to the design of online learning resources and classroom learning activities because these are the basis of the flipped classroom. In the past, many teachers completely relied on existing course teaching materials without any updates. Now they need to develop all or part of the teaching materials and classroom learning activities each time before the new courses start. In this study, a flipped course model suitable for research methods courses was extended based on previous research.

In addition, based on the uncertainty of the post-pandemic era, the implementation of courses combined online and offline teaching. In this study, in accordance with the requirements of governmental units for school pandemic prevention measures, the mixed teaching design was carried out online first and then offline to present the real situation. More specifically, in the 8-week course planning of this study, online teaching was used for the first 4 weeks, and offline teaching was used for the last 4 weeks. In the post-pandemic era, studies designed based on real situations are more helpful to understand the educational effects in the real field.

Hong et al. (2022) stated that the belief-action-outcome (BAO) model can explain the relationship between people’s beliefs, behaviors, and outcomes in educational research. Therefore, according to the framework of this model, in this study, four relevant constructions were chosen to construct the model according to the literature review to examine how students’ personal beliefs affected their learning behavior in the blended flipped classroom in the post-pandemic scenario. The relative discussions are presented in the following paragraphs.

### Self-efficacy

Self-efficacy is an individual’s belief in their ability to perform a specific task (Bandura, 1977). People reflect different levels of belief according to different tasks and domains. Furthermore, Wofford (2021) proposed that self-efficacy could predict self-confidence. Zhen et al. (2017) indicated that academic self-efficacy had a motivating effect on learning tasks. In this study, the effect of self-efficacy in a specific field on the learning process and outcomes (self-confidence) of the flipped classroom was discussed.

### Learning engagement

Learning engagement is defined as the degree to which students contribute to an activity, and this variable is considered a key element of learning activities (Huang et al., 2022). If students are motivated and focused on the learning process, they are likely to persevere in their learning (Padgett et al., 2019). In a learner-centered teaching activity, it is necessary to understand the engagement of the participants in the course. Therefore, in this study, this variable was used to examine students’ participation in the course.

### Cognitive load

Cognitive load theory has long been recognized as having a major impact on the field of educational psychology. One of the main guidelines of the theory is that extraneous cognitive load must be reduced and learners’ working memory should not be overloaded in order to leave sufficient cognitive resources for actual learning (Skulmowski and Xu, 2021). Cognitive load theory suggests that the purpose of teaching is to allow learners to store large amounts of domain-specific information in their long-term memory so they can work effectively in a variety of contexts in the future (Darejeh et al., 2021). Since cognitive load influences learners’ interactions with each other in the learning environment, this can lead to optimizing or hindering information processing in working memory (Bahari, in press). Therefore, this study examined the impacts of cognitive load on learning processes and outcomes.

### Self-confidence

Self-confidence is widely recognized as an important asset in helping individuals succeed (Gelaidan and Abdullateef, 2017). Self-confidence is considered as a state that varies as tasks are completed on time (Fan and Ye, 2022) and is therefore used as a learning outcome (Olaussen et al., 2020). Blended learning can effectively enhance students’ self-confidence (Bouilheres et al., 2020). In addition, Anthony et al. (2019) proposed that emphasis should be placed on enhancing the learner’s self-confidence in blended learning. Since self-confidence is a very important learning outcome in the learning process, this study used it as a dependent variable.

### Research purposes

In the post-pandemic era, blended learning in flipped classrooms should be a feasible response, but there are few empirical studies on how learners’ beliefs affect their behavior and outcomes in such courses. Therefore, in order to effectively expand the understanding of this topic, this study examined the relationship between students’ academic self-efficacy, learning engagement, cognitive load, and enhancement of academic self-confidence (EASC) in the context of blended learning in a flipped classroom.

## Theoretical framework and research hypothesis

### Belief-action-outcome theory

The belief-action-outcome (BAO) model proposed by Melville (2010) effectively explains individual behaviors and final outcomes. When beliefs are accepted by individuals, individuals are driven to justify and defend their beliefs (Ojo et al., 2019, 2020). In other words, beliefs influence an individual’s behavior in terms of sustainability. In sum, the BAO framework helps not only to understand the antecedents of beliefs, but also to theorize how these beliefs influence actions (Molla et al., 2014). BAO is widely used in research in the field of science and technology. In recent years, it has been extensively adopted by Ho et al. (2020) to explain the relationship between learners’ beliefs, actions, and outcomes in educational research. As a result, the BAO model was adopted in this study to explain how the participants’ ability beliefs affected their learning actions and the final learning outcomes obtained from this experiment.

### Research model

**FIGURE 1:**
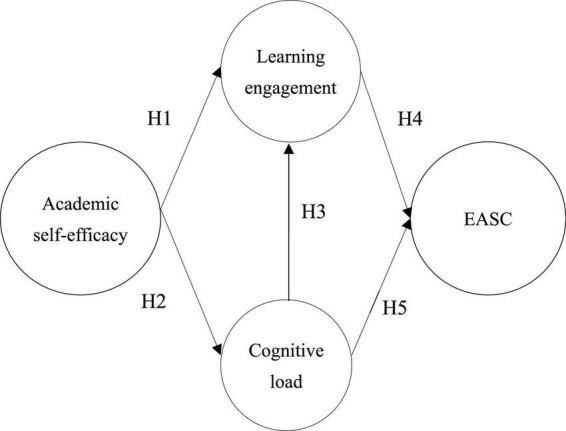
Research model.

The BAO model is a research model that can be used to explain learner behavior and to illustrate the relationship between people’s ability beliefs, learning actions, and subsequent outcomes in the field of education (Hong et al., in press). Therefore, based on the BAO framework, this study proposed five research hypotheses and constructed a research model to determine four constructs, namely academic self-efficacy (belief), cognitive load, learning engagement (action), and EASC (outcome). The relationship between the constructs is shown in Figure 1.

### Research hypothesis

#### The relationship between academic self-efficacy and learning engagement

Beliefs have a significant impact on an individual’s behavior (Molla et al., 2014), as those with lower perceptions of self-efficacy tend to avoid special tasks, while those who perceive themselves as competent are more likely to engage in them (Artino, 2012). For example, people’s ability beliefs about the knowledge of one particular subject may influence the ways in which they study the subject (Rosman et al., 2017). In other words, self-efficacy also influences behavior (Vasile et al., 2011). Alamri (2022) found that learners with strong academic self-efficacy boosted their learning engagement in a variety of learning environments such as e-learning. In addition, Zhen et al. (2017) found that academic self-efficacy has a motivating effect on learning tasks, and students with high academic self-efficacy are more actively involved in their learning (Azila-Gbettor and Abiemo, 2021). Based on the above findings, this study assumed that students were more engaged in blended learning when they had higher levels of self-efficacy. Therefore, this study examined the relationship between participants’ academic self-efficacy and their learning engagement. The hypothesis is as follows:

H1: Academic self-efficacy has a positive impact on learning engagement.

#### The relationship between academic self-efficacy and cognitive load

In an academic environment, students’ levels of self-efficacy are thought to affect their cognitive abilities (Bandura et al., 1996). A strong sense of self-efficacy will generate a strong sense of competence and then promote cognitive processes (Vasile et al., 2011). In learning environments, high cognitive load tasks are more susceptible to cognitive distraction when self-efficacy is threatened (Vancouver and Kendall, 2006). When students have high self-efficacy, the cognitive load in the learning process can be alleviated (Wahyuni et al., 2020). Studies have confirmed that self-efficacy is negatively correlated with cognitive load (Zheng et al., 2009). Besides, the research of Hong et al. (2020a) also confirmed that self-efficacy in a specific academic field can affect the cognitive load in innovative teaching. When participants have higher self-efficacy, their cognitive load tends to be lower (McQuaid, 2010; Redifer et al., 2021). Based on the above, this study assumed that students with higher levels of self-efficacy would be less overloaded with learning information when they received the blended instruction. Therefore, this study examined the relationship between participants’ academic self-efficacy and their cognitive load. The hypothesis is as follows:

H2: Academic self-efficacy has a negative impact on cognitive load.

#### The relationship between cognitive load and learning engagement

Cognitive load affects visual attention and behavior (Papavlasopoulou et al., 2018). In other words, cognitive load affects students’ learning engagement (Bradford, 2011; Oluwajana et al., in press). Atiomo (2020) clearly pointed out that excessive cognitive load can significantly affect learning engagement. The research of Altinpulluk et al. (2020) also confirmed that when the cognitive load of distance learners is reduced, the students’ engagement in the learning process is increased. Other studies have also found that students with higher cognitive load tend to have lower levels of engagement (Dong et al., 2020). Based on the above, this study examined the relationship between participants’ cognitive load and learning engagement. The hypothesis is as follows:

H3: Cognitive load has a negative impact on learning engagement.

#### The relationship between learning engagement and enhancement of academic self-confidence

Learning engagement refers to how active students participate in effective educational practice and how they commit to educational goals and learning. High-quality learning engagement is considered to be closely related to academic success (Skinner and Pitzer, 2012). As time passes, self-confidence can be stimulated and maintained if one’s behaviors lead to definite outcomes (Clark and Chalmers, 1998). As a result, learning engagement is considered highly cohesive to achieving learning outcomes (Elshami et al., 2022). In fact, learners’ engagement has a direct impact on student success and performance, and it has been proven that students’ learning engagement has a causal relationship with success. If students do not actively participate in and are dissatisfied with the learning environment, it is difficult to obtain positive and effective learning outcomes (Almasri et al., 2021; Almasri, 2022). Hence, students’ learning engagement helps to build up their self-confidence when they are learning seriously (Jacobs, 2020). The research of Hong et al. (in press) also confirmed that learning engagement is positively correlated with EASC. Based on these findings, this study inferred that when students received blended learning, the better their learning engagement or behaviors, the more EASC they would experience. Therefore, this study examined the relationship between participants’ learning engagement and EASC. The hypothesis is as follows:

H4: Learning engagement has a positive impact on EASC.

#### The relationship between cognitive load and enhancement of academic self-confidence

Cognitive load is how learners interact with each other in the learning environment, and causes optimization or hinders information processing in working memory (Bahari, in press). In fact, complex learning content will reduce learners’ learning efficiency, which is the fundamental reason for the inability to achieve effective teaching (Li, 2022). Besides, the experience of cognitive overload can lead to feelings of frustration or failure (McQuaid, 2010). Cognitive theory plays an important role in helping students understand self-confidence (Siregar et al., 2020); as a result, poor learning due to cognitive load affects self-confidence. The experience of success in challenging tasks increases students’ self-confidence (Koumi, 2013). However, self-confidence fluctuates dynamically and is affected by cognitive load (Gottlieb et al., 2022). Studies have found that cognitive load is negatively correlated with EASC (Hong et al., 2017). Based on the findings, this study assumed that when students received excessive learning information during the blended learning courses, it might inhibit their EASC. Therefore, this study examined the relationship between participants’ cognitive load and EASC. The hypothesis is as follows:

H5: Cognitive load has a negative impact on EASC.

## Research method

This study adopted a single-group quasi-experimental design method to conduct teaching experiments, since most of the time it is difficult to conduct randomized controlled trials in educational settings (Gopalan et al., 2020). A single-group quasi-experimental design is considered as an effective research method that helps to solve the problem when there are too few participants or it is not possible to implement traditional experimental designs (Fan and Ye, 2022). This study used a quasi-experimental design method to conduct this blended learning research based on a flipped classroom design.

### Course design

#### Course model

The flipped classroom is an innovative and effective teaching method that transfers the course content taught by teachers in class to the preschool stage using multimedia, thus allowing students to master their own learning progress (Lin and Hwang, 2019). During the class time, teachers can mainly focus on helping students identify knowledge, eliminate knowledge blind spots, develop problem-solving skills, and promote cooperation among students (Angadi et al., 2019). In the flipped classroom, teachers no longer teach in the form of old-fashioned traditional lectures, but play the role of knowledge facilitators to guide students to construct knowledge independently.

After unifying and integrating many existing flipped classroom models, Guo (2019) proposed a general O-PIRTAS flipped classroom model, including objective, preparation, instructional video, review, test, activity, and summary in a total of seven stages. This model was confirmed to be effective in terms of promoting the learning outcomes of college students, and it is considered as a suitable Chinese localized flipped classroom approach. However, the flipped classroom is an evolving teaching method in higher education (Day, 2018), so it is still necessary to continuously improve or develop effective course models.

In addition, the concept of knowledge transfer is often mentioned in relation to the purpose and meaning of academic programs, because students with knowledge transfer ability can clearly understand their own learning process and more easily apply knowledge acquired in one environment to another (Humrickhouse, 2021). Hong et al. (2020b) also suggested that teachers can freely transfer stages to encourage students to think about similarities and applications of what they have learned. Therefore, a transfer stage was added to the flipped classroom model in this study.

In the course, the knowledge gained from each stage was used to facilitate students’ reflection and communication throughout the learning process (Al-Samarraie et al., 2020), and it was a process of discussion between teachers and students in order to facilitate a shift from thinking to practice (Levett-Jones, 2005), because highly autonomous learners can engage more actively in learning activities by asking questions and participating in discussions (Geng et al., 2019). Therefore, a discussion stage was added to the flipped classroom model in this study.

**FIGURE 2:**
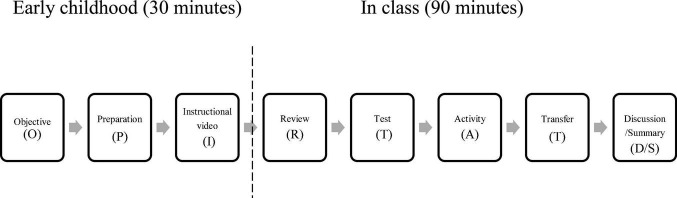
O-PIRTATD/S model.

To sum up, based on the O-PIRTAS model, this study added two stages: transfer (T) and discussion (D), and extended the flipped classroom model to the O-PIRTATD/S model, as shown in Figure 2. The O part was for students to understand the primary learning objectives (knowledge) and advanced learning objectives (competence) of the course; the P part was to carry out course preparation activities; the I part was to watch the teaching video; the R part was to review the content of pre-course activities; the T part was to have a classroom pop-quiz/test; the A part was to carry out classroom activities; the T part was to transfer knowledge; and the D/S part was teacher-student discussion and teacher’s summary.

#### Course content

Teaching materials are the basis of teaching activities and the concrete embodiment of curriculum content (Tien, 2006). In all educational stages in China, teaching materials are an important basis for curriculum design; this is also the case in higher education. Teaching materials serve the function of providing the teaching bases and have a definite effect on the teaching content, teaching plan, and course progress (Zhao, 2021). Not only can teaching materials affect the quality of teaching and the cultivation of students’ abilities, but they can also promote teaching reform (Wang and Shan, 2022). The curriculum content design of this study is based on Zhu (2019) book, “Scientific Research Methods in Preschool Education.” This book discusses the concepts and specific research processes of preschool education research (Zhou, 2021). Since the vast majority of the participants in this study did not have a knowledge base of research methodology, the content of this study was divided into eight units arranged and designed according to the chapters of this book, allowing participants to construct knowledge from shallow to deep. The content is shown in Supplementary Appendix 1.

### Procedure

This study conducted a teaching experiment in the spring semester of 2022. After planning this study, a proposal was made to the leadership of an education college in Guangxi, China. With the consent of the administration of the school, three classes of students taking the research methods course at that time were invited to participate in this study. The participating students were informed that they could decide whether or not to participate in this study, the data collected during the research process would be kept confidential, and the results of participating in the experiment would not affect their grades. After giving informed consent, all the students in the three classes agreed to participate in this study.

Before the formal class of this study started, the learning significance of the O-PIRTATD/S flipped classroom was explained to the participating students. In the following 8 weeks of the study, a recorded teaching video was provided to the students before each class to conduct flipped learning. In the first 4 weeks of the course, online teaching was conducted through Tencent Conference (one of the most well-known conference software packages in China), and offline teaching was conducted physically in the classrooms for the next 4 weeks.

At the end of the course in the 8th week, the instructor provided a link to the online questionnaire for students to fill in. This institute used Wenjuanxing (one of the most commonly used platforms in China) to make the online questionnaire. To minimize the tendency of participants to fill out the questionnaire to meet the social expectations, they were asked to be completely honest while answering. Finally, it was ensure that the information collected would be kept completely anonymous and confidential.

### Participants

A total of 133 students participated in this study with a total of 133 questionnaires collected. After 18 incomplete questionnaires were deleted, 115 valid data (86.5% data availability) were usable. Among the participants, there were eight (6.9%) male students and 107 (93.1%) female students; those with previous flipped classroom experience numbered 16 (13.9%), and those without numbered 99 (86.1%); those with mixed learning experience before the epidemic numbered 10 (8.7%) and those with no blended learning experience numbered 105 (91.3%). The mean age of the participants was 21.84 years old, and the standard deviation was 0.93 years. Although the gender ratio in this study had a huge gap, it still fit with the findings of Nong et al. (2022), who stated that in the early childhood education environment, the majority of practitioners are female, which causes a significant gender ratio difference.

Westland (2010) also pointed out that the sample size requirement was still a thorny issue in SEM-based research, and the minimum standard of sample size in SEM research could be found according to the following formula: *n* 50*r*^2^ − 450*r* + 1100, where n is the number of samples and r is the number of construct items of latent variables. The research in this study was based on the formula proposed by Westland. Among the potential variables in this study, the minimum number of items was four, so r was set to 4 in the formula: “*n* 50*r*^2^ − 450*r* + 1100.” The calculation showed “n ≥ 100,” so at least 100 valid samples were needed to meet Westland’s requirements. In sum, the valid data for this study numbered 115, which met the recommended requirement.

### Measurement

This study was based on the analysis of the results of the questionnaire survey on students’ learning experience. The content of the questionnaire was modified from a research tool with good reliability and validity, and was reviewed by three university professors with Ph.D. degrees to ensure its content validity. The scale design of the questionnaire content was based on a 5-point Likert scale, with the meaning of the values ranging from 1, strongly disagree, to 5, strongly agree.

#### Academic self-efficacy

This study referred to and modified the hands-on efficacy scale of Hong et al. (in press) with a total of eight questions to measure whether participants assessed their confidence and ability to perform this research task when undertaking a university dissertation topic. For example, “As long as I work hard, I will be able to solve the problems encountered in the research of the graduation thesis” and “When I encounter difficulties in the research of the graduation thesis, I will draw up a variety of solutions.” This original questionnaire had a Cronbach’s alpha value of 0.88 and a factor loading (FL) value of 0.74.

#### Learning engagement

This study used the Learning Engagement Scale from Carmona-Halty et al. (2019) with a total of nine questions to measure the participants’ perceptions of their participation in this blended learning based on the O-PIRTATD/S flipping model. Questions included: “I feel energized and competent while studying or in class” and “I am immersed in my studies.” This original questionnaire had a Cronbach’s alpha of 0.90 and a FL of 0.74.

#### Cognitive load

This study referred to and revised the Intrinsic Cognitive Load Scale of Hong et al. (2019) with a total of six questions to measure the participants’ assessment of their feelings of degree of load about the course content in this blended learning based on the O-PIRTATD/S flipped model. For example, “I find the content of the research methods class difficult and it is quite strenuous for me” and “I have to pay a lot of attention to keeping up with the questions asked by the teacher.” The Cronbach’s alpha value of the original questionnaire was 0.85, and the FL value was 0.72∼0.79.

#### Enhancement of academic self-confidence

This study used the Academic Confidence Scale of Chang et al. (2022) with a total of seven questions to measure whether participants assessed whether they had perceived an increase in self-confidence in conducting research. For example: “After finishing the course, I feel that I am getting better at planning and research design, so I am confident in my ability to complete my dissertation” and “After finishing the course, I feel that I have found more and more innovative research topics, so I am confident in my ability to complete the thesis.” The Cronbach’s alpha of the original questionnaire was 0.79, and the FL was 0.63∼0.76.

## Results and discussion

This study used SPSS for reliability and validity analysis, and then used the SEM method to verify the research model with AMOS to test the relationship between academic self-efficacy, learning engagement, cognitive load, and EASC. A complete verification and results of the analysis are presented as follows.

### Item analysis

The item analysis of this study confirmed the degree of fit of each construct. Each fit index and recommended value, including the χ^2^/df value, should be less than 5; the root mean square error of approximation (RMSEA) should be less than 0.10; the goodness of fit index (GFI) and the adjusted goodness of fit index (AGFI) should be higher than 0.80; items with factor loadings (FL) not higher than 0.50 should be deleted from the original questionnaire (Kenny et al., 2015; Hair et al., 2019), as shown in Table 1. The number of questions in the construct of academic self-efficacy was reduced from eight to six; the number of questions in the construct of learning engagement was reduced from nine to six; the number of questions in the construct of cognitive load was reduced from six to four; and the number of questions in the construct of EASC was reduced from seven to four.

**TABLE 1:** First-order confirmatory analysis.

Index	Critical value	Academic self-efficacy	Learning engagement	Cognitive load	EASC
χ^2^	—	14.60	11.70	2.60	0.64
*df*	—	9	9	2	2
χ^2^/*df*	<5	1.62	1.31	1.3	0.32
RMSEA	<0.10	0.07	0.05	0.05	0.00
GFI	>0.80	0.96	0.97	0.99	0.99
AGFI	>0.80	0.91	0.93	0.94	0.99
FL	>0.50	0.74∼0.91	0.66∼0.86	0.52∼0.84	0.60∼0.84
*t*	>3	10.20∼13.26	9.28∼12.60	7.94∼11.68	7.95∼11.72

In this study, the external validity of the items was used to determine the scope of interpretation of the study (Cor, 2016). The values of all respondents for each item were divided into the top 27% and the bottom 27%, and a *t*-test was performed; if the t value was greater than 3 (^***^p < 0.001), the external validity was considered to be at a significant level. However, Table 1 showed that the t-values of the constructs ranged from 7.94 to 13.26 (^***^p < 0.001), which meant that all items in this study had acceptable external validity (Green and Salkind, 2004).

### Reliability and validity analysis

Before carrying out the path analysis, the reliability and validity of each measurement model (variable) should be checked. In this study, the Cronbach’s alpha was used to confirm the internal consistency of the test scale, and the composite reliability (CR) was used to test the reliability. Hair et al. (2019) suggested that if Cronbach’s alpha was higher than 0.70, the value was regarded as an acceptable standard. He also suggested that the CR value should exceed the standard of 0.70. The Cronbach’s alpha value and CR value in this study were both between 0.80 and 0.91 which met the recommended standards, as shown in Table 2.

**TABLE 2:** Reliability and validity analysis.

Construct	M	SD	α	CR	AVE	FL
	—	—	>0.70	>0.70	>0.50	>0.50
Academic self-efficacy	2.44	0.62	0.91	0.91	0.64	0.80
Learning engagement	2.46	0.67	0.89	0.89	0.58	0.76
Cognitive load	2.70	0.69	0.80	0.80	0.51	0.70
EASC	2.48	0.56	0.82	0.83	0.56	0.74

Convergent validity was judged by factor loading (FL) and average variance extracted (AVE). Hair et al. (2019) pointed out that the FL value should be higher than 0.50, and the questions below this value should be deleted. All the questions retained in this study met the standards recommended by scholars, among which the FL value was between 0.70 and 0.80, as shown in Table 2. Hair et al. (2011) suggested that the AVE value must be greater than 0.50 to indicate that the construct has convergent validity. The AVE values were between 0.51 and 0.64, as shown in Table 2.

Confirming that each variable is independent can avoid bias in statistical results. Awang (2015) indicated that the AVE root value of each construct is greater than the Pearson correlation coefficient of other constructs, which means that the construct has discriminant validity. The analysis results showed that each construct in this study had discriminant validity, as shown in Table 3.

**TABLE 3:** Discriminant validity analysis.

Construct	1	2	3	4
Academic self-efficacy	(0.89)			
Learning engagement	0.63	(0.81)		
Cognitive load	−0.40	−0.37	(0.83)	
EASC	0.73	0.62	−0.40	(0.74)

The value on the diagonal is the square root value of AVE, and the other values are the correlation coefficient values.

### Model fit analysis

Passing the model fit analysis is a prerequisite for model validation and path analysis. Each fitness index and recommended value including χ^2^/df value must be less than 5 (Hair et al., 2019); the RMSEA value should be less than 0.10; GFI, AGFI, NFI, NNFI, CFI, IFI, and RFI should be greater than 0.80 (Abedi et al., 2015), while PNFI and PGFI equivalents should be greater than 0.50 (Hair et al., 2019). The fitted index values for this study were χ^2^ = 238.4, df = 165, χ^2^/df = 1.45, RMSEA = 0.06, GFI = 0.84, AGFI = 0.80, NFI = 0.84, NNFI = 0.94, CFI = 0.94, IFI = 0.94, RFI = 0.82, PNFI = 0.73, and PGFI = 0.66. Based on the above results, the model was fit for the examination.

### Path analysis

**FIGURE 3:**
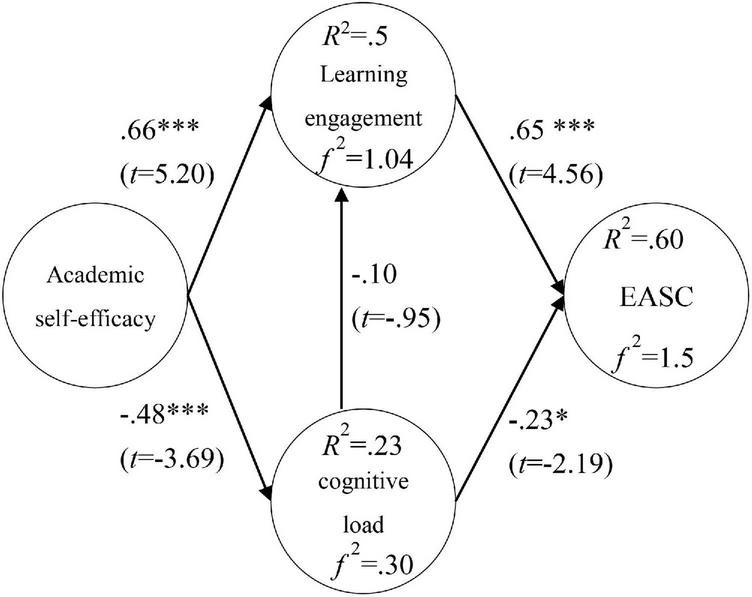
Research model validation. *p < 0.5, ***p < 0.001.

Path analysis results showed that academic self-efficacy had a positive impact on learning engagement (β = 0.66^***^; *t* = 5.20), and a negative impact on cognitive load (β = −0.48^***^; *t* = 3.69); Cognitive load had no significant effect on learning engagement (β = −0.10; *t* = −0.95); learning engagement had a positive effect on EASC (β = 0.65^***^; *t* = 4.56); cognitive load had a negative effect on EASC (β = −0.23*; *t* = −2.19), as shown in Figure 3. In addition, the explanatory power of academic self-efficacy to learning engagement was 51%, *f*^2^ was 1.04; the explanatory power of academic self-efficacy to cognitive load was 23%, *f*^2^ was 0.30; and the explanatory power of learning engagement and cognitive load to EASC was 60% and *f*^2^ was 1.5, as shown in Figure 3.

### Discussion

#### Academic self-efficacy has a positive impact on learning engagement

The results of this study proved that academic self-efficacy had a positive impact on learning engagement. In other words, when students have higher levels of self-efficacy, they will be more engaged in blended learning. This result is also in line with the research findings of Zhen et al. (2017), Azila-Gbettor and Abiemo (2021), and Alamri (2022). Academic self-efficacy has a motivating effect on learning tasks, and students with high academic self-efficacy will be more actively involved in their learning. It could be said from the above that in the mixed teaching situation based on flipped classrooms, students’ self-efficacy is one of the important prerequisites for promoting students’ participation in classroom activities.

#### Academic self-efficacy has a negative impact on cognitive load

The results of this study showed that academic self-efficacy had a negative impact on cognitive load. That is to say, when students have higher levels of self-efficacy, they are less overloaded with processing learning information when they receive blended learning instruction. This also confirmed the findings of Buchner et al. (2022). The findings indicated that prioritizing teaching is conducive to the acquisition and application of knowledge, which can improve self-efficacy in specific areas and reduce cognitive loads. Moreover, Wahyuni et al. (2020) confirmed that when students have high self-efficacy, the cognitive load in the learning process can be alleviated. In addition, Zheng et al. (2009) found that self-efficacy was negatively correlated with cognitive loads. Besides, McQuaid (2010) and Redifer et al. (2021) also confirmed that when participants’ self-efficacy is fulfilled, cognitive load tends to be reduced. Based on the above, in the mixed teaching situation based on the flipped classroom, the level of students’ self-efficacy affected their cognitive processing and absorption load in the learning process.

#### Cognitive load has no significant effects on learning engagement

The results of this study showed that cognitive load had no significant effect on learning engagement, which is different from the results of previous studies. It can be concluded that when students receive blended learning, the learning information they receive in a timely manner makes them feel a sense of load, but it will not reduce their investment in this learning method. This may be explained by Kester et al. (2011), who argued that when managing overall cognitive load to facilitate learning, the irrelevant load must first be eliminated. They explained that the cognitive load related to the learning task has a lower negative impact on learning engagement, unlike the cognitive load unrelated to the learning content which will have a more serious impact. Additionally, as explained by Likourezos and Kalyuga (2017), the learners’ engagement and motivation is increased if the cognitive load is imposed by complex problem-solving tasks prior to teaching, which in turn compensates for high cognitive demands. Therefore, learners have a moderate cognitive load in the learning process, which will not have a negative impact on their learning engagement. This indicated that students’ cognitive load could not predict cognitive load in the mixed teaching situation based on the flipped classroom teaching style.

#### Learning engagement has a positive impact on enhancement of academic self-confidence

The results of this study showed that learning engagement had a positive impact on the enhancement of academic self-confidence. In sum, when students receive blended learning, the better their engagement behaviors, the more enhanced their academic self-confidence will be. The results of this study were also consistent with previous research findings; for example, Skinner and Pitzer (2012) pointed out that learning engagement refers to students’ active participation in effective educational practice and their commitment to educational goals and learning. In addition, Almasri et al. (2021) and Almasri (2022) further proposed that learner engagement has a direct impact on students’ academic success, and a causal relationship has been demonstrated. At the same time, Jacobs (2020) proposed that students’ high level of engagement in learning will help build their self-confidence. Hong et al. (2021) also confirmed that engagement is positively related to the enhancemet of self-confidence. Based on the above, if students are not actively engaged in the learning environment and feel dissatisfied with it, it is difficult for them to obtain positive learning outcomes.

#### Cognitive load has a negative impact on enhancement of academic self-confidence

The results of this study showed that cognitive load had a negative impact on EASC. In sum, when students receive excessive and irrelevant cognitive information when receiving blended learning, it may hinder their EASC. This result is also in line with the viewpoints of past studies; for example, Bahari (in press) pointed out that cognitive load which can advance or hinder information processed in working memory affects how learners interact with each other in the learning environment. In addition, Siregar et al. (2020) proposed that cognitive theory plays an important role in helping students understand self-confidence. For example, McQuaid (2010) confirmed that excessive cognitive load experience can lead to feelings of frustration or failure. A high level of cognitive load creates a poor learning experience for the learner. Moreover, Hong et al. (2017) found that cognitive load was negatively correlated with self-confidence enhancement. In sum, in the mixed teaching situation based on the flipped classroom, the more cognitive load students have, the more disadvantageous it will be to promoting their self-confidence.

## Conclusion and suggestions

### Conclusion

How to provide an effective and flexible teaching method is an important issue in the post-pandemic era. Under the circumstances of the post-pandemic era, this study adopted a single-group quasi-experimental design method in a research methods course taken by undergraduate students majoring in Early Childhood Education in a university in China. This study implemented O-PIRTAS/D-based online and offline teaching for 4 weeks each with the blended flipped learning approach.

The features of the expanded flipped curriculum model are that students participating in the flipped classroom course must know the learning objectives of the course, prepare for the classes, watch the videos to complete the self-learning tasks before class, and complete the tests well when they are in class. Student participation requires mutual cooperation in groups to carry out the learning activities. Teachers guide students to think about the application of knowledge viewpoints in different situations, so they can have a high degree of interaction after knowledge transfer. Before the end of the course, the teacher summarizes the knowledge viewpoints of the course, and creates a highly learner-centered learning situation through the above-mentioned classroom stages. This curriculum model for blended learning is one of the contributions of this research.

Moreover, from the perspective of the BAO model, this study examined the relationship between academic self-efficacy, learning engagement, cognitive load, and EASC. The study results showed that: 1. academic self-efficacy has a positive impact on learning engagement and a negative impact on cognitive load; 2. cognitive load has no significant impact on learning engagement; 3. learning engagement has a positive impact on EASC; and 4. cognitive load has a negative impact on EASC.

In addition, the BAO model was mainly used in the field of green energy technology in the past, and it has rarely been used in the educational research field. This study is the first to use the BAO model and its theory to explain students’ learning behavior in classroom activities. The validity of the BAO model as a theoretical framework to construct a model of learning behavior is confirmed, which helps to expand the understanding of this theory in the field of teaching and is an important contribution of this study.

Due to the city’s anti-pandemic policy for schools, the schools cooperating with the government offered online courses for the first 4 weeks and then offline courses next. One of the peculiarities of this study is the course design of 4-week blended learning based on a real situation, which also allowed learners to participate in the research in the most realistic situation, which can avoid as much as possible the occurrence of the Hawthorne effect.

### Suggestions

The results of this study show that self-efficacy is an important antecedent variable for learning. When students have good self-efficacy, it helps to increase their learning engagement, while also inhibiting their cognitive load. Therefore, how to enable learners to develop a high level of self-efficacy is an important issue. The research methods course is very important for students in China, and a prerequisite course that needs teachers’ strong teaching skills. This study suggests that before teachers formally teach research methods courses, teachers can use educational practice cases or small scientific research activities to explain, so that students will not feel that research is an unattainable and difficult task to establish students’ belief in their ability to conduct research.

Learner-centered learning has been widely recognized as an important way to provide students with meaningful learning experiences. In this study, it was confirmed that in the blended learning, the use of the flipped model based on O-PIRTASD/S reduced learners’ excessive cognitive load, and their cognitive load in this flipped classroom model did not affect their learning. As a result, it is suggested that teachers can use the O-PIRTASD/S flipped model for instructional design, so students can deeply participate in the course and play the leading role in learning activities.

### Limitations and suggestions for future research

Based on the results, most of the assumed paths between constructs were confirmed. When participants have low levels of ability beliefs and learning engagement, they still cannot acquire good learning outcomes. However, this study was a confirmatory study, and the reasons behind it cannot be explained from the data results, which also explains why the participants’ perceptions of each aspect remain unknown. It is very important to understand participants’ either positive or negative perspectives on flipped classrooms and blended learning and the reasons for low learning engagement in future research. This issue can be examined in more detail through qualitative research methods including in-depth interviews to formulate appropriate academic counseling strategies.

This study adopted a quasi-experimental design method to ensure a realistic classroom situation, but it was limited by the number of participants. Although this research method has been proved to be an effective and suitable method in teaching research, the participants were not randomly assigned, which leads to problems inferring to other contexts. In the future follow-up research, it is suggested that more students from different departments be recruited to carry out mixed teaching research based on flipped classrooms to more extensively verify the applicability of the extended flipped classroom model and research model in different contexts. At the same time, a two-group quasi-experimental design can also be carried out; that is, the teaching effect of the experimental group and the control group can be compared to confirm the effect of this teaching method.

In an educational sense, many things may cause participants to feel and think differently depending on the contexts. The post-pandemic era is a complicated situation. For learners or educators, their views, motivations, or acceptance of online learning or blended learning may change due to the pandemic. In the future follow-up research, situational expectancy-value theory (SEVT) can be taken into consideration to discuss the perceptions of learners or teachers on the same learning task in different contexts, thereby expanding the understanding of the influence of situational factors.

## Data availability statement

The raw data supporting the conclusions of this article will be made available by the authors, without undue reservation.

## Ethics statement

Ethical review and approval was not required for the study on human participants in accordance with the local legislation and institutional requirements. Written informed consent for participation was not required for this study in accordance with the national legislation and the institutional requirements.

## Author contributions

WN and J-HY: concept and design, drafting of the manuscript, acquisition of data, and statistical analysis. WN, J-HY, PC, and Y-SL: critical revision of the manuscript. All authors contributed to the article and approved the submitted version.
