# Supplementary material for: A study on the blended learning effects on students majoring in preschool education in the post-pandemic era: An example of a research-method course in a Chinese university
Source: Front Psychol. 2023 Jan 11;13:962707. doi: 10.3389/fpsyg.2022.962707 (PMC9874681; doi:10.3389/fpsyg.2022.962707)
Supplement: Supplementary file 1 [file Table_1.doc]

**Appendix 1.**

Course Content Planning

| **Week** | **Content** | **Objectives** |
| --- | --- | --- |
| 1 | Basic concepts of educational research | Primary learning objective: understand the definitions, principles, and types of educational research  Advanced learning objective: learn to distinguish reliability and validity, horizontal research, and longitudinal research; simply design horizontal and longitudinal studies according to the topic |
| 2 | The function and research path of educational research | Primary learning objective: understand the function and research path of educational research  Advanced learning objective: learn to distinguish between quantitative research and qualitative research, and simply design quantitative research and qualitative research according to the topic |
| 3 | Basic concepts of preschool education research | Primary learning objective: understand the definition, purpose, research objects, and hot issues of preschool education research  Advanced learning objective: learn to distinguish the difference between preschool educational research and educational research, analyze and master the scope of specific hot issues in preschool education research, and migrate to other educational research objects according to preschool education research objectives |
| 4 | Basic steps in preschool education research | Primary learning objective: understand the basic steps of preschool research  Advanced learning objective: learn the basic steps of analyzing the research of others and designing research based on a prescribed theme |
| 5 | Research ethics in preschool education research | Primary learning objective: understand the ethical principles of preschool education research and the definitions and types of professional ethics in scientific research Advanced learning objective: through the video case of the experiment, the research ethics are analyzed through the video case of the experiment, and the general ethical code of educational research is transferred to the code of ethics with young children as the research object |
| 6 | Research questions | Primary learning objective: understand the principles of choosing research questions, sources and writing conventions, and research significance  Advanced learning objective: determine whether the statement is appropriate and correct according to the title, learn to formulate research questions |
| 7 | Literature review | Primary learning objective: understand the definition of literature review and types of literature, and understand the role of literature review in educational research Advanced learning objective: master the process and methods of literature retrieval, and be able to write a preliminary literature review. |
| 8 | Sampling | Primary learning objective: understand the definition of population, sample and element, and be familiar with the concept and classification of random sampling and non-random sampling  Advanced learning objective: be able to distinguish between random sampling and non-random sampling, and choose the appropriate sampling method according to the research object and research purpose |
